# Meditope–Fab interaction: threading the hole

**DOI:** 10.1107/S2053230X17016272

**Published:** 2017-11-18

**Authors:** Krzysztof P. Bzymek, Yuelong Ma, Kendra N. Avery, David A. Horne, John C. Williams

**Affiliations:** a Department of Molecular Medicine, Beckman Research Institute of City of Hope, 1710 Flower Street, Duarte, CA 91101, USA

**Keywords:** meditope, monoclonal antibodies, X-ray crystallography, surface plasmon resonance

## Abstract

Meditope, a cyclic 12-residue peptide, binds to a unique binding side between the light and heavy chains of the cetuximab Fab. In an effort to improve the affinity of the interaction, it was sought to extend the side chain of Arg8 in the meditope, a residue that is accessible from the other side of the meditope binding site, in order to increase the number of interactions. These modifications included an *n*-butyl and *n*-octyl extension as well as hydroxyl, amine and carboxyl substitutions. The atomic structures of the complexes and the binding kinetics for each modified meditope indicated that each extension threaded through the Fab ‘hole’ and that the carboxyethylarginine substitution makes a favorable interaction with the Fab, increasing the half-life of the complex by threefold compared with the unmodified meditope. Taken together, these studies provide a basis for the design of additional modifications to enhance the overall affinity of this unique interaction.

## Abstract 2

The structure–affinity relationship of complexes of the cetuximab Fab with meditope peptides modified at Arg8 is investigated.

## 1. Introduction   

Monoclonal antibodies (mAbs) are central components in the diagnosis, imaging and treatment of cancers and autoimmune diseases, among other diseases (Weiner *et al.*, 2012 ▸; Baker & Isaacs, 2017 ▸). Their broad applicability stems from their high target specificity and desirable pharmacokinetics (Kamath, 2016 ▸). ‘Naked’ mAbs inhibit ligand binding of the target receptor and block cell signaling, lead to down-regulation of receptors on the cell surface, recruit other components of the immune system through antibody-dependent cell-mediated cytotoxicity (ADCC) or complement-dependent cytotoxicity (CDC), or a combination of all three (Bakema & van Egmond, 2014 ▸; Stasiłojć *et al.*, 2016 ▸). Their specificity has also led to the development of antibody–drug conjugates, where they act as targeting moieties to deliver cytotoxic cargo to the site of the disease. Currently, this is achieved through direct chemical conjugation of a payload to the targeting antibody (Trail & Bianchi, 1999 ▸; Wu & Senter, 2005 ▸).

**Figure 1:**
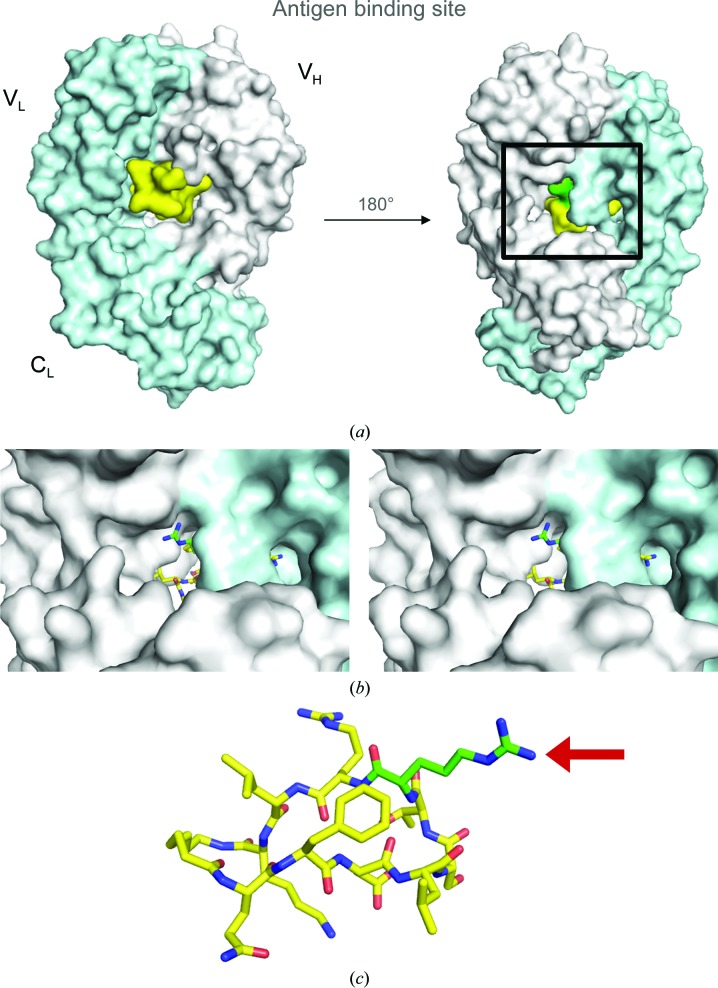
(*a*) Meditope-binding site in the cetuximab Fab. The light chain is shown in light blue, the heavy chain in dark blue and the meditope is shown in yellow; Arg8 is highlighted in green. (*b*) Stereo image of the back side of the cetuximab Fab with meditope residues shown as yellow and green sticks. (*c*) The structure of the aminoheptanoic acid (AHA)-linked meditope (Bzymek, Ma *et al.*, 2016 ▸); modifications of NH_2_ of the guanidinium group of Arg8 (highlighted in green) are the subject of this report.

Our recent discovery of meditope, a cyclic peptide that binds to a Fab cavity of cetuximab, can serve as an alternative, noncovalent method to modify antibodies, with a precise ratio of two meditopes to one IgG. To use this interaction for delivery, we have focused on enhancing the lifetime of the interaction. In previous efforts, we have focused on increasing the affinity by altering the cyclization as well as altering the side chains of the meditope (Bzymek, Avery *et al.*, 2016 ▸; Bzymek, Ma *et al.*, 2016 ▸). We have also determined important residues emanating from the Fab through systematically grafting the binding site onto M5A, an anti-CEA mAb (Zer *et al.*, 2017 ▸). In this report, we investigate the possibility of increasing the affinity of this interaction by reaching through to the other side of the Fab hole (Fig. 1 ▸).

## 2. Materials and methods   

Meditope peptides were synthesized at the Synthetic and Biopolymer Chemistry Core (City of Hope, Duarte, California, USA) following established procedures (Bzymek, Avery *et al.*, 2016 ▸; Bzymek, Ma *et al.*, 2016 ▸). The general method for the synthesis of meditope derivatives substituted at Arg8 is based on a previously published procedure (Martin & Liskamp, 2008 ▸).

**Figure 2:**
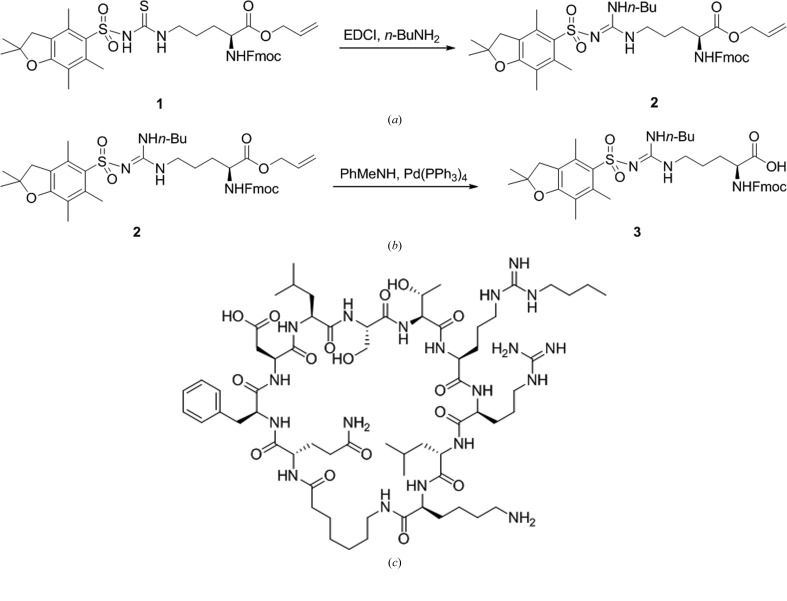
Method for the synthesis of meditope derivatives substituted at Arg8. (*a*) Synthesis of **2** from **1**. (*b*) Synthesis of **3** from **2**. (*c*) *n*-Butyl-substituted Arg8 meditope prepared according to a standard solid-phase Fmoc synthesis protocol using **3**.

Briefly, to a stirred solution of **1** (Fig. 2 ▸ *a*; 23 mg, 0.03 mmol) in dichloromethane (0.6 ml) were added EDCI (12 mg, 0.06 mmol, two equivalents) and *n*-butylamine (4.4 mg, 0.06 mmol, two equivalents). After 5 min at room temperature, the solvent was removed in vacuum. The residue was purified by silica-gel column chromatography (40–50% ethyl acetate/hexane) to afford the product **2** (23 mg, 95%).

To a stirred solution of **2** (Fig. 2 ▸ *b*; 23 mg, 0.03 mmol) in THF (0.8 ml) were added *N*-methylaniline (10 mg, 0.09 mmol, three equivalents) and Pd(PPh_3_)_4_ (2 mg, 0.0015 mmol, 0.05 equivalents). After 45 min at room temperature, the solvent was removed in vacuum. The residue was purified by silica-gel column chromatography (25:1:0.1 methanol:dichloromethane:acetic acid) to afford the product **3** (20 mg, 90%).

Additionally, the *n*-butyl-substituted Arg8 meditope (Fig. 2 ▸ *c*) was prepared according to a standard solid-phase Fmoc synthesis protocol using the Fmoc-*N*-butyl Arg derivative **3**.

All other meditope derivatives substituted at Arg8 were synthesized using the same method as above. All peptides were purified using reverse-phase HPLC (Agilent 1200 system with an Agilent Prep-C18 column, 21.2 × 150 mm, 5 µm) with a water (0.1% TFA)/acetonitrile (0.1% TFA) solvent system. All peptides were characterized by mass spectrometry.

The cetuximab Fab was prepared as described previously (Donaldson *et al.*, 2013 ▸). Cetuximab Fab–meditope complexes were crystallized by the hanging-drop vapor-diffusion method. The Fab was mixed with excess meditope (1:10 to 1:14 molar ratio of cetuximab Fab:meditope), and precipitant (1.4–1.8 *M* amonium sulfate, 0.1 *M* sodium citrate pH 4–6) was added to give a final ratio of 1:1 protein–meditope:precipitant.

Crystals of the complexes of cetuximab with meditopes were passed through mother liquor with 20–25% propylene glycol and cooled in a cryostream. Diffraction data were collected on a Rigaku MicroMax-007 HF with an R-AXIS IV++ detector at 100 K and processed with *XDS* (Kabsch, 2010 ▸) followed by refinement using *PHENIX* (Adams *et al.*, 2010 ▸) as described previously (Bzymek, Ma *et al.*, 2016 ▸). All structures have been deposited in the RCSB PDB (http://www.rcsb.org): cetuximab Fab–meditope complexes (AHA)QFDLST*X*RLK, where *X* is *N*-(*n*-butyl)arginine, PDB entry 6au5; *X* is *N*-(3-hydroxypropyl)arginine, PDB entry 6azk; *X* is *N*-(3-aminopropyl)arginine, PDB entry 6ayn; *X* is *N*-(carboxyethyl)arginine, PDB entry 6azl; *X* is *N*-(*n*-octyl)­arginine, PDB entry 6axp.

All surface plasmon resonance (SPR) experiments were performed on a GE Biacore T100 instrument (GE Healthcare) at 25°C as described previously (Bzymek, Avery *et al.*, 2016 ▸; Bzymek, Ma *et al.*, 2016 ▸). Briefly, cetuximab IgG ligand was amine-coupled to CM5 chips using acetate buffer pH 5.5 at a density of 5000 RU. Analytes were prepared in GE buffer HBS-EP+ [10 m*M* HEPES pH 7.4, 150 m*M* NaCl, 3 m*M* EDTA, 0.05%(*v*/*v*) surfactant P20]. Kinetics experiments were carried out at a flow rate of 30 µl min^−1^ using HBS-EP+ as both the running and regeneration buffer. Experimental data were processed using *Biacore T100 Evaluation* software v.2.0.1. Purified peptides were dissolved in water and extensively dialyzed to remove any residual TFA, and their concentration was determined as described previously (Bzymek, Ma *et al.*, 2016 ▸). Note that all reported off-rates are independent of peptide concentration.

## 3. Results and discussion   

**Figure 3:**
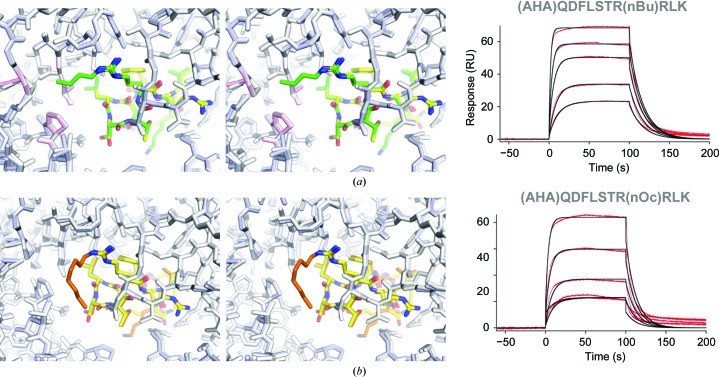
Stereo images of short modifications of Arg8 superimposed on the AHA-linked meditope (yellow) with the corresponding SPR sensograms. (*a*) *n*-Butyl extension of Arg8 (green) does not result in overall changes to the meditope, but threads through to the other side of the cetuximab Fab. Pro9, Leu114 and Pro155 HC are highlighted in light red. (*b*) The *n*-octyl extension (brown) of Arg8 points away from the Fab and positions the chain between Pro155 and Thr157 in the heavy chain and Asn41 in the light chain.

Our recent efforts to characterize and optimize the affinity of the meditope–Fab complex focused on the importance of cyclization (Bzymek, Ma *et al.*, 2016 ▸) and side-chain modifications (Bzymek, Avery *et al.*, 2016 ▸). In these structural analyses, we observed that the guanidinium nitrogen (NH_2_) of arginine in position 8, located in the ‘back’ of the meditope-binding pocket, is partially exposed to the other side of the Fab (Fig. 1 ▸). Of note, there are two copies of Fab–meditope complexes in the asymmetric unit. We wondered whether it was possible to reach through the other side of the Fab hole by extending the side chain of Arg8, increasing the surface area to improve the affinity of the meditope–Fab interaction. To test this, we synthesized a series of extensions of Arg8 starting with the addition of aliphatic carbon chains. All modifications were introduced into the meditope with an aminoheptanoic acid (AHA) linker. Initially, we synthesized a relatively short, *n*-butyl extension to determine whether such a construct would make favourable interactions with the cetuximab Fab. We observed that this construct bound to the cetuximab Fab (Fig. 3 ▸ *a*, Tables 1 ▸ and 2 ▸) with comparable kinetics to the respective aminoheptanoic acid (AHA)-linked meditope (*K*_d_ = 2.7 and 1.8 µ*M*, respectively). The methyl group extended towards a hydrophobic pocked lined with Pro9, Leu114 and Pro155 HC. We wondered whether extending the *n*-butyl group by four methylene groups, to an *n*-octyl group, would increase the hydrophobic surface area and result in productive interactions with the Fab. The long aliphatic chain of *n*-octyl­arginine is well ordered (the average *B* factor of the eight C atoms is only ∼30% higher than that for the side chain of Arg8) and packs between the ring of Pro155 and Thr157 HC on one side and Asn41 LC on the other (Fig. 3*b* ▸). However, the affinity was further reduced (*K*_d_ values of 8.5 and 1.8 µ*M* for the *n*-octyl Arg8 meditope and the original AHA-linked meditope, respectively; Fig. 3 ▸ *b* and Table 2 ▸), with the on-rate and the off-rate being affected to a similar extent.

**Figure 4:**
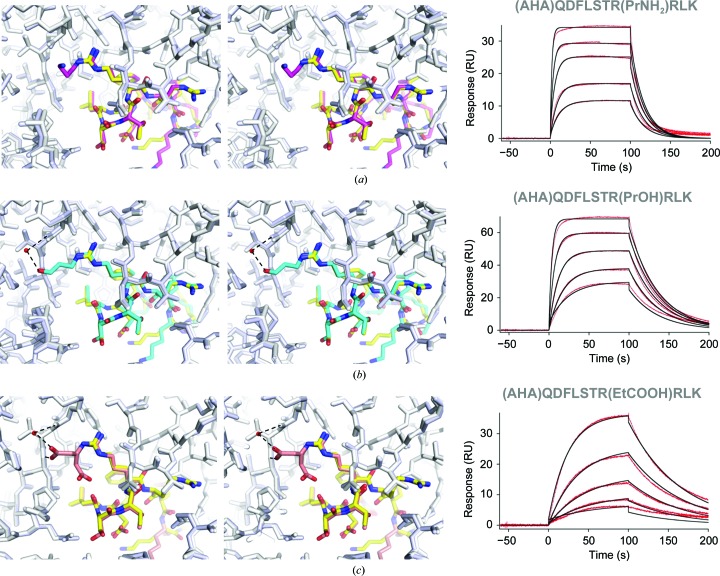
Modifications of the methyl group of *n*-butyl Arg8 shown in stereo. The AHA-linked meditope is shown in yellow. Corresponding SPR traces are shown on the right. (*a*) No productive interactions are observed upon modification with an amine. (*b*) Replacing the *n*-butyl methyl group with a hydroxyl Arg8 (cyan) results in coordination of a water molecule (red sphere) and decreases the off-rate. (*c*) A carboxyethyl modification (light red) of Arg8 appears in multiple conformations, one of which is involved in similar interactions to the hydroxypropyl extension, whereas the second points away from the Fab and towards the meditope backbone.

**Figure 5:**
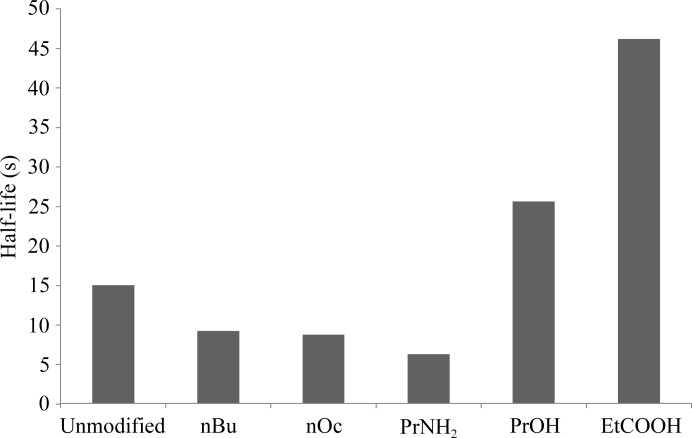
Half-lives of Arg8 variant meditope–cetuximab interactions calculated using the formula *t* = ln(2)/*k*_d_. The off-rate of bimolecular interactions is independent of peptide/Fab concentration. All data were collected at 25°C in HBS-EP+ buffer pH 7.4. Unmodified, AHA-linked meditope with no modifications to Arg8; nBu, *N*-(*n*-butyl)arginine variant; nOc, *N*-­(*n*-octyl)arginine variant; PrNH_2_, *N*-(3-aminopropyl)arginine variant; PrOH, *N*-(3-hydroxypropyl)arginine variant; EtCOOH, *N*-(carboxy­ethyl)arginine variant.

Given that the addition of the extended hydrophobic groups did not improve the affinity, we investigated the possibility of adding polar groups that can participate in hydrogen bonds. We observed in the structure that the terminal methyl group is in close proximity to the backbone carbonyl of Gly112 HC (3.3 Å for the first copy of the meditope in the asymmetric unit and 3.5 Å for the second) and the backbone amide of Leu114 HC (∼3.3 Å); thus, we expected that modification of this group with a hydrogen-bond donor or acceptor could improve the affinity. To test this hypothesis, we synthesized and solved structures of three analogs with polar groups: an amine (3-aminopropyl; Fig. 4 ▸ *a*), a hydroxyl group in place of the methyl group (3-hydroxypropyl; Fig. 4 ▸ *b*), and a carboxyethyl extension (Fig. 4 ▸ *c*). In each case we preserved the original length of the main chain of the *n*-butyl group (four atoms). Substitution with the amine analog reduced the affinity approximately threefold (*K*_d_ values of 9.2 and 2.7 µ*M* for the amine analog and *n*-butyl analog, respectively; Table 2 ▸), which based on the structure may be attributed to a lack of productive interactions of the terminal amine with the Fab (Fig. 4 ▸ *a*); in fact the amine group is facing away from the Fab scaffold and into the solvent. A hydroxyl in the same position binds with a similar dissociation constant (*K*_d_ values of 2.3 and 2.7 µ*M* for 3-hydroxypropyl and *n*-butyl, respectively) and a slower off-rate (*k*_d_ value of 0.027 s^−1^ for 3-hydroxypropyl compared with 0.060 s^−1^ for the *n*-butyl variant). The crystal structure indicates that the terminal hydroxyl group forms a hydrogen bond to the Fab. The distance of the 3-hydroxy­propyl O atom from the backbone amide of Leu114 LC is ∼2.8 Å, and it is in close proximity to a water molecule (*d*_OH⋯HOH_ = 2.8 Å), which also forms a hydrogen bond to the backbone carbonyl O atom of Gly112 LC (2.8 and 2.9 Å for the two copies of the meditope in the asymmetric unit; Fig. 4 ▸ *c*). These interactions are likely to result in a slower off-rate (longer half-life) compared with the 3-aminopropyl and *n*-butyl analogs (Fig. 5 ▸ and Table 2 ▸).

Similarly, the slower off-rate for the carboxyethyl derivative of Arg8 (*k*_d_ = 0.015 s^−1^; Table 2 ▸) may be explained by the formation of hydrogen-bonding interactions between one of its O atoms and the peptide backbone of Leu114 LC (2.9 and 3.2 Å; Fig. 4 ▸ *c*). This slower off-rate may reflect a more favourable interaction at the pH used for the Biacore assay (the SPR pH was 7.4 and that of the mother liquor was 5.5). At a pH of ∼5.5 the carboxyl group may be partially protonated and act as a hydrogen-bond donor. The electron-density maps indicate the presence of two side-chain rotamers. In one rotamer, the carboxyl moiety is pointing towards the backbone of the meditope and is within hydrogen-bonding distance of the carbonyl O atom of Leu5 (*d*_C=O⋯HOOC_ = 3.0 Å for one copy of the meditope in the asymmetric unit and 3.6 Å for the other). The second rotamer is in a similar position to the hydroxyl group in the 3-hydroxypropyl Arg8 meditope. Not unexpectedly, the bridging water molecule observed in the 3-hydroxypropyl Arg8 meditope structure described above is also present and is involved in analogous interactions (the Arg8 carboxyethyl oxygen–water distance is 2.8 and 3.1 Å for the two copies of the meditope in the asymmetric unit, and *d*_C=O⋯HOH_ for the peptide backbone O atom of Gly112 LC is 2.9 and 3.1 Å for the two copies of the meditope). The overall affinity of this analog is lower compared with the corresponding AHA-linked meditope largely owing to a much slower on-rate (*k*_a_ values of 0.13 × 10^4^ and 2.5 × 10^4^ *M* ^−1^ s^−1^, respectively).

In summary, increasing the surface area and adding groups that are capable of forming electrostatic interactions between the meditope and the meditope-enabled mAb improved the half-life of the interaction. The simple extension of the Arg8 with an *n*-butyl group indicated that it is possible to thread the Fab ‘hole’ (Fig. 1 ▸). While hydrophobic modifications of Arg8 (*n*-butyl and *n*-octyl aliphatic extensions) slightly reduced the half-life of the complex, the addition of hydrogen-bond acceptors (in the 3-hydroxyproline and carboxyethyl extensions) resulted in 1.7-fold and 3-fold increases in the half-life, respectively, over the nonmodified AHA-cyclized meditope (Fig. 5 ▸). Combining the extensions of Arg8 with modifications at positions 3 and/or 5 of the meditope (Bzymek, Avery *et al.*, 2016 ▸), and of the linker region (Bzymek, Ma *et al.*, 2016 ▸), is expected to further improve the affinity and half-life of the interaction.

## Supplementary Material

PDB reference: cetuximab with amino­heptanoic acid-linked *n*-butylarginine meditope variant, 6au5

PDB reference: with aminoheptanoic acid-linked *N*-(3-aminopropyl)-l-arginine meditope variant, 6ayn

PDB reference: with aminoheptanoic acid-linked *N*-(3-hydroxypropyl)-l-arginine meditope variant, 6azk

PDB reference: with aminoheptanoic acid-linked *N*-carboxyethylarginine meditope variant, 6azl

PDB reference: with aminoheptanoic acid-linked *n*-octyl­arginine meditope variant, 6axp

## Figures and Tables

**Table 1:** Data-collection and refinement statistics Values in parentheses are for the highest resolution shell.

	(AHA)QFDLSTR(nBu)RLK (PDB entry 6au5)	(AHA)QFDLSTR(PrNH_2_)RLK (PDB entry 6ayn)	(AHA)QFDLSTR(PrOH)RLK (PDB entry 6azk)	(AHA)QFDLSTR(EtCOOH)RLK (PDB entry 6azl)	(AHA)QFDLSTR(nOc)RLK (PDB entry 6axp)
Data collection					
Space group	*P*2_1_2_1_2_1_	*P*2_1_2_1_2_1_	*P*2_1_2_1_2_1_	*P*2_1_2_1_2_1_	*P*2_1_2_1_2_1_
Unit-cell parameters					
*a*, *b*, *c* (Å)	64.58, 83.06, 212.83	64.40, 82.90, 212.00	64.36, 82.93, 212.05	64.57, 83.05, 212.11	64.39, 82.94, 212.34
α, β, γ (°)	90.0, 90.0, 90.0	90.0, 90.0, 90.0	90.0, 90.0, 90.0	90.0, 90.0, 90.0	90.0, 90.0, 90.0
Resolution (Å)	33.17–2.48 (2.55–2.48)	33.12–2.48 (2.54–2.48)	33.00–2.48 (2.67–2.60)	33.17–2.48 (2.55–2.48)	33.12–2.48 (2.55–2.48)
Wilson *B* factor (Å^2^)	37.8	30.2	28.9	29.9	30.2
*R*_meas_	0.038 (0.165)	0.066 (0.296)	0.067 (0.207)	0.032 (0.103)	0.051 (0.214)
CC_1/2_	0.999 (0.973)	0.998 (0.921)	0.998 (0.954)	0.999 (0.990)	0.995 (0.956)
〈*I*/σ(*I*)〉	34.8 (9.2)	24.3 (5.3)	19.8 (6.5)	42.3 (14.0)	31.8 (7.1)
Completeness (%)	98.0 (85.0)	99.0 (91.0)	98.8 (90.1)	99.3 (92.2)	99.6 (96.2)
Multiplicity	5.5 (4.3)	4.6 (3.5)	4.8 (3.5)	5.4 (3.7)	5.9 (4.2)
Refinement					
Resolution (Å)	2.48	2.48	2.48	2.48	2.48
No. of reflections	40272	40766	40779	40961	40882
*R*_work_/*R*_free_ (%)	18.6/22.7	17.2/22.2	17.0/21.3	17.0/21.6	17.9/22.8
No. of atoms					
Fab	6577	6623	6596	6561	6577
Meditope	184	193	202	200	192
Water	294	458	526	431	481
*B* factors (Å^2^)					
Fab	38.3	24.9	22.6	24.8	25.1
Meditope	50.7	33.9	26.1	30.7	32.6
Water	37.3	29.7	27.9	30.6	31.6
R.m.s.d.s					
Bond lengths (Å)	0.005	0.007	0.005	0.008	0.008
Bond angles (°)	1.023	0.896	1.077	1.168	1.200
Ramachandran					
Favored/allowed/disallowed	96.8/3.0/0.2	97.1/2.9/0.0	96.9/3.1/0.0	97.9/2.1/0.0	97.3/2.7/0.0

**Table 2:** Binding kinetics of meditope variants to cetuximab at 25°C All experiments were performed at pH 7.4, unless noted otherwise.

Meditope	*k*_a_ (*M* ^−1^ s^−1^) × 10^4^	*k*_d_ (s^−1^)	*K*_d_ (µ*M*)
CQFDLSTRRLKC†	8.8	0.015	0.17
(AHA)QFDLSTRRLK	2.5	0.046	1.8
(AHA)QFDLSTRRLK at pH 5.5	2.0	0.075	3.8
(AHA)QFDLSTR(nBu)RLK	2.2	0.060	2.7
(AHA)QFDLSTR(nOc)RLK	0.93	0.079	8.5
(AHA)QFDLSTR(PrNH_2_)RLK	1.2	0.110	9.2
(AHA)QFDLSTR(PrOH)RLK	1.2	0.027	2.3
(AHA)QFDLSTR(EtCOOH)RLK	0.13	0.015	11.8
(AHA)QFDLSTR(EtCOOH)RLK at pH 5.5	0.06	0.020	32.0

† Data from Bzymek, Ma *et al.* (2016 ▸).
